# Comment on ‘bibliometric insights into systemic sclerosis with renal involvement: trends, contributions, and future directions’

**DOI:** 10.1080/0886022X.2025.2486563

**Published:** 2025-04-11

**Authors:** Yiran Chen, Jingyuan Zhang

**Affiliations:** a First Affiliated Hospital of Dalian Medical University, Liaoning, Dalian, China; b Dalian Medical University, Liaoning, Dalian, China

Dear Editor,

The article entitled ‘Bibliometric insights into systemic sclerosis with renal involvement: trends, contributions, and future directions’ by Haochen huang et al. is interesting [1]. It is commendable that the disclosure of data supplements and completeness in this document are amazing. A long-term concern in the field of bibliometrics is that only the retrieval strategy is disclosed, but the specific data are difficult to reproduce, including the possibility that different institutions have different access rights to the available documents in the same database. In addition, the research scope of this study is the literature in the field from 2000 to 2024. The database of the Web of Science Core Collection was improved after 1997, while other databases, such as Scopus, were fully improved after 2011. The confusion in database selection may lead to confusion regarding the judgment of frontier landscapes in the field. For example, if the documents included in the first decade of the twenty first century are mainly based on Scopus databases and data from the WOSCC database are added, the data from the latter will lead to a sharp increase in the intensity of keyword outbreaks, but it is not in line with the actual situation; on the other hand, it will lead to the weakening of some keywords. In short, for different databases, it is difficult to truly reflect the actual situation of the research field based on the combination of different processing environments without correction.

However, the interpretation and supplementation of some data are still worth improving. For example, the explanation of the dual-map overlay of journals in this study is not sufficient. The knowledge flow between different fields is not uniform but heterogeneous [2]. The author has standardized the knowledge flow relationship based on citation frequency through Z index [3]. For example, the Z index of knowledge flow in the field between ‘MEDICINE, MEDICAL, CLINICAL’ and ‘MOLECULAR, BIOLOGY, GENETICS’ is 4.216238, ‘MOLECULAR, BIOLOGY’, The z index of the domain flow between ‘IMMUNOLGY’ and ‘MOLECULAR, BIOLOGY and GENETICS’ is 3.8252413, and the corresponding citation frequency is 8370. Other data were difficult to obtain because of the occlusion of the figures in the original research picture processing process. The acquisition of these data needs to thank the journal ‘Renal Failure’ for its high-quality requirements for manuscript pictures.

Additionally, some data are worth disclosing. For example, the original research considers that different topics in the field are divided based on thematic maps made using bibliometrix. As the author stated, this interpretation needs to be combined with a comparison of the density and centrality of each theme, but the specific data are not disclosed. In addition, some statistical standards should be supplemented; for example, the bibliometrix [4] platform provides a variety of clustering algorithms, as well as the number of keywords included in the thematic map drawing process, the frequency of the minimum set, and the minweightindex index, etc. The disclosure of these indicators can deeply understand the conclusions of this study, and further prove the reliability of the author’s conclusions based on scientometrics analysis in this research field from the interpretation of quantitative indicators. Simultaneously, it provides complete and sufficient evidence for researchers interested in this research field to help them judge the frontier in the field and make decisions on the choice of their future research direction.
